# Benefits and Challenges in Using Seroprevalence Data to Inform Models for Measles and Rubella Elimination

**DOI:** 10.1093/infdis/jiy137

**Published:** 2018-03-19

**Authors:** Amy K Winter, Micaela E Martinez, Felicity T Cutts, William J Moss, Matt J Ferrari, Amalie McKee, Justin Lessler, Kyla Hayford, Jacco Wallinga, C Jess E Metcalf

**Affiliations:** 1 Department of Ecology and Evolutionary Biology, Princeton University, Princeton, New Jersey; 2 Department of Environmental Health Sciences, Mailman School of Public Health, Columbia University, New York, New York; 3 London School of Hygiene and Tropical Medicine, London, United Kingdom; 4 Department of Epidemiology, Johns Hopkins Bloomberg School of Public Health, Baltimore, Maryland; 5 International Vaccine Access Center, Department of International Health, Johns Hopkins Bloomberg School of Public Health, Baltimore, Maryland; 6 Center for Infectious Disease Dynamics, The Pennsylvania State University, State College, Pennsylvania; 7 Netherlands National Institute for Public Health and the Environment, Bilthoven, Netherlands; 8 Leiden University Medical Center, Leiden, Netherlands

**Keywords:** elimination, measles, rubella, serology, serosurvey

## Abstract

**Background:**

Control efforts for measles and rubella are intensifying globally. It becomes increasingly important to identify and reach remaining susceptible populations as elimination is approached.

**Methods:**

Serological surveys for measles and rubella can potentially measure susceptibility directly, but their use remains rare. In this study, using simulations, we outline key subtleties in interpretation associated with the dynamic context of age-specific immunity, highlighting how the patterns of immunity predicted from disease surveillance and vaccination coverage data may be misleading.

**Results:**

High-quality representative serosurveys could provide a more accurate assessment of immunity if challenges of conducting, analyzing, and interpreting them are overcome. We frame the core disease control and elimination questions that could be addressed by improved serological tools, discussing challenges and suggesting approaches to increase the feasibility and sustainability of the tool.

**Conclusions:**

Accounting for the dynamical context, serosurveys could play a key role in efforts to achieve and sustain elimination.

## Teaser

We use simulations to highlight how (accounting for the dynamical context) high-quality measles and rubella serological surveys can be used to inform key control and elimination questions if the challenges of conducting, analyzing, and interpreting them are overcome.

**(See the Editorial commentary by Durrheim, on pages 341–3.)**

Infectious diseases can persist in populations if there are enough individuals susceptible to infection to acquire and transmit infection. Infection from fully immunizing pathogens, such as measles and rubella viruses, leads to lifelong immunity, thus depleting the number of susceptible individuals in a population. Therefore, pathogen persistence is only possible if susceptible individuals are replenished via births and immigration of susceptible persons. Measles and rubella vaccines are highly effective, reducing the rate of accumulation of susceptible individuals. These vaccines provide indirect protection to susceptible individuals by reducing the probability of effective contact between infectious and susceptible individuals, in addition to direct protection of immunized individuals. As a result, successful immunization programs can curtail or eliminate pathogen transmission [1]. More importantly, a small proportion of individuals fail to develop a long-lasting immune response after vaccination [2, 3]: thus, we refer to “vaccination” as the administration of vaccine and “immunization” as the induction of a protective immune response via vaccination.

High measles vaccination coverage worldwide has reduced measles incidence and mortality to low levels in most countries, although progress in achieving high coverage with the first dose of measles-containing vaccines (MCVs) has slowed recently [4]. Although MCVs have been in widespread global use for over 40 years, rubella-containing vaccines (RCVs) have only recently been introduced into low-income countries [5]. The World Health Organization (WHO) Region of the Americas was certified as achieving elimination of endemic rubella and measles in 2015 and 2016, respectively [6]. The remaining 5 WHO regions have measles elimination targets, and 3 have set rubella control or elimination targets for 2020 [5]. Elimination efforts include attaining and sustaining high coverage with the first dose of MCV, scaling up of routine vaccination with a second dose of MCV, introduction of RCVs, and supplemental immunization activities ([SIAs] or campaigns) of both MCV and RCV [5].

Mathematical models indicate that elimination requires the achievement of a threshold level of population immunity (ie, the percentage of the population immune). This threshold differs between measles and rubella and between settings, but it is determined by the pathogens’ transmission potential [1] and the birth rate [7]. Elimination must be maintained by sustaining this level of population immunity through immunization or preventing reintroduction of the virus. The WHO recommends that programs use good quality data to monitor population immunity by identifying and responding to large numbers of susceptible individuals [8].

Although analytic techniques can be used to infer population immunity profiles from vaccination coverage [9] and incidence data [10], these methods necessarily rely on indirect inference of immunity rather than direct measurement. Vaccination coverage data are often of poor quality [11] and can misrepresent population immunity because most countries lack data on vaccine effectiveness under field conditions and, assuming an average vaccine effectiveness, may overestimate population immunity in countries with weak cold chain systems. Furthermore, where multiple doses are offered (eg, a routine second dose or SIAs), doses may be disproportionately delivered to individuals who received a first dose [12]. Disease surveillance data may be biased due to nonspecific diagnosis, preferential reporting of disease in young children, and gross underreporting (eg, in 2016, WHO estimated approximately 7 million (95% confidence interval, 4.2–28.7 million) global cases [4], but only 132137 were reported to the WHO [13]). For rubella, reporting is even less sensitive because surveillance was introduced recently, and 20%–50% of rubella cases are subclinical or asymptomatic [14].

Serology can provide a direct measure of population immunity. After infection or immunization, pathogen-specific immunoglobulin (Ig)M and IgG antibodies are produced. Immunoglobulin M antibodies persist for a few weeks, whereas IgG antibodies persist for years to decades, although levels may decline over time. Measles virus-specific and rubella virus-specific IgG antibodies are recognized correlates of immunity, and antibody concentrations in the blood exceeding a threshold are deemed protective against infection or disease [15, 16]. Thus, in principle, high-quality serological surveys, ie, cross-sectional household surveys in which these antibodies are measured, allow direct measurement of a population’s immunological profile. Depending on their design, serological surveys can reveal “immunity gaps” (ie, age groups or spatial locations where immunity is lower than expected, or below some operational threshold), thus identifying areas for additional vaccination efforts [17].

Because natural and vaccine-derived immunity cannot be distinguished, interpreting serological data requires accounting for historical changes in disease incidence and vaccination coverage (achieved both through routine services and SIAs), as we illustrate in this paper. We first delineate expectations for age-specific immunity profiles across a spectrum from endemicity to elimination. We describe how inferring immunity profiles from vaccination coverage data and/or reported case data (the current method used by most countries) can result in biases, showing the potential added value of serology. We then describe how serology can be used to address 2 key questions for the control and elimination of measles and rubella: (1) what are the most effective vaccination strategies to control and eliminate infection, and (2) how effective are current vaccination programs? We conclude by discussing challenges of serological surveys and provide suggestions for improving their feasibility and sustainability.

## METHODS

### Age-Specific Serological Profiles: Expectations Across a Transmission Spectrum From Endemic to Elimination

**Figure 1.**
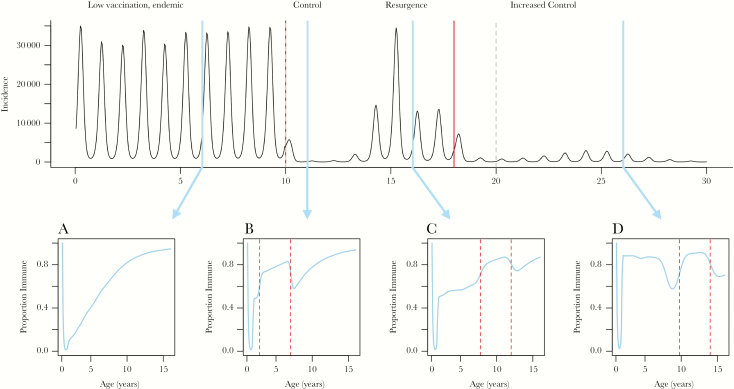
A simulated population from a rubella transmission model (see Supplementary Materials S1 for model details). Top panel displays the time series of incidence of infection in a changing context of increased vaccination coverage (see Table 1). Bottom panels A–D display age profiles of immunity after 6, 11, 16, 26 years, respectively; dashed red vertical lines indicate age ranges affected by the supplemental immunization activities (SIAs) in preceding years. Bottom panel shows (A) proportion immune under low vaccination coverage results in a gradual increase over age after the decay of maternal immunity. (B) Increased routine vaccination to 50% slows the rate of acquisition of immunity through natural infection, but vaccine-acquired immunity increases at rates reflecting routine vaccination delivery and SIAs, resulting in further age-specific increases in targeted age groups (affecting 2- to 7-year-olds here). (C) During periods of control, immunity in relevant age classes reflects routine vaccination coverage in ages 1 to 6 years, corresponding to low incidence between year 10 and year 13 (see incidence time series). In year 15, a resurgence affects individuals just outside the target age group of the first SIA, ie, individuals older than age 6 in year 10, and older than age 12 in year 16. (D) Erratic age-specific immunity profiles emerge under increased routine coverage: although immunity in ages 1–6 closely reflects the 90% vaccination coverage, the dip in 8-year-olds occurs because the second SIA reduced incidence, and therefore these children had a low risk of natural infection but were too young to be immunized during the second SIA, or the increase in routine coverage that occurred in year 20.

**Figure 2.**
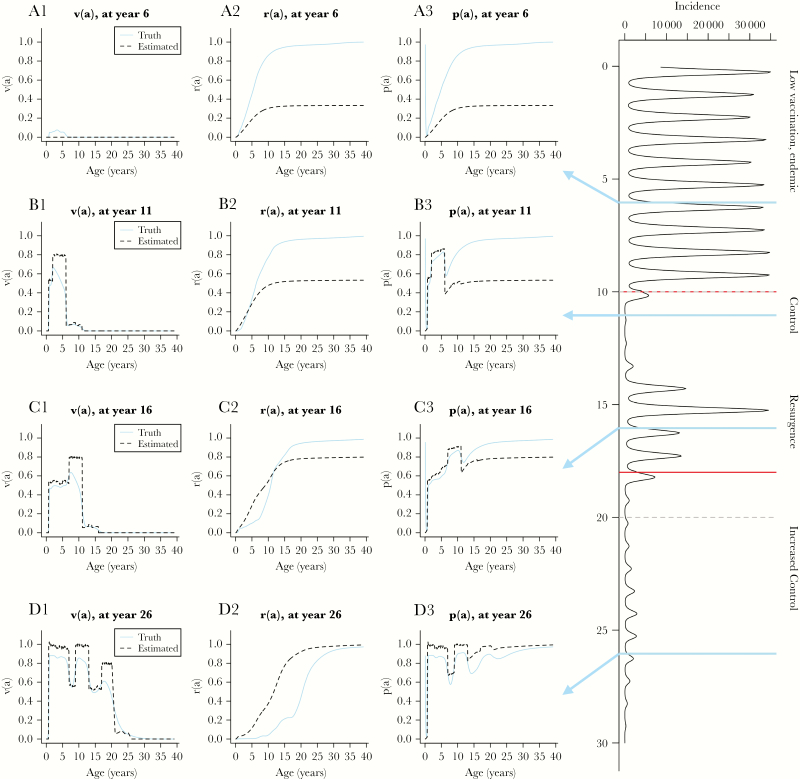
Comparison of inferred age-specific immunity from inaccurate coverage and case surveillance data to “true” age-specific immunity using a simulated population. Right panel displays time series of incidence in a changing context of increased vaccination coverage (see top panel, Figure 1). Left panels A1–D3 display age-specific proportion vaccinated (v(a); A1, B1, C1, and D1), proportion recovered from natural infection (r(a); A2, B2, C2, and D2), and proportion immune (p(a); A3, B3, C3, and D3) at years 6, 11, 16, 26, respectively. Blue lines represent the “truth”, and black dashed lines represent the inferred age trajectories, assuming some error in vaccination and incidence data (see Table 2 for data error assumptions). False positives from estimated v(a) and false negatives from estimated r(a) both contribute to the error in estimated p(a). The added value of a high-quality representative immunoglobulin G serological data (assuming minimal diagnostic testing error) is clear because uncertainties associated with vaccination coverage and incident case data greatly bias estimates of age-specific immunity.

Seropositivity in young infants results from the transplacental transfer of maternal IgG antibodies to the fetus. Antibody levels then decay exponentially as the infant ages. The proportion of seropositive children then increases at a rate determined by the rate of immunization through vaccination or infection. Age-specific seroprevalence profiles vary over time because vaccine coverage and infection transmission vary, as described below and illustrated in Figures 1 and 2 (model assumptions are described in Tables 1 and 2, methods are decribed in Supplementary Materials S1).

**Table 1.** Assumed Routine and SIA Vaccination Coverage Over Time in the Simulated Population Displayed in Figure 1^a^

Year	Routine Coverage (Age Range) Red Solid Lines in Figure 1	SIA Coverage (Age Range) Gray Dashed Lines in Figure 1
0	~10% (9–12 mo)	
1	~10% (9–12 mo)	
2	~10% (9–12 mo)	
3	~10% (9–12 mo)	
4	~10% (9–12 mo)	
5	~10% (9–12 mo)	
6	~10% (9–12 mo)	
7	~10% (9–12 mo)	
8	~10% (9–12 mo)	
9	~10% (9–12 mo)	
10	~50% (9–12 mo)	70% (1–5 yo)
11	~50% (9–12 mo)	
12	~50% (9–12 mo)	
13	~50% (9–12 mo)	
14	~50% (9–12 mo)	
15	~50% (9–12 mo)	
16	~50% (9–12 mo)	
17	~50% (9–12 mo)	
18	~50% (9–12 mo)	80% (1–5 yo)
19	~50% (9–12 mo)	
20	~90% (9–12 mo)	
21	~90% (9–12 mo)	
22	~90% (9–12 mo)	
23	~90% (9–12 mo)	
24	~90% (9–12 mo)	
25	~90% (9–12 mo)	
26	~90% (9–12 mo)	
27	~90% (9–12 mo)	
28	~90% (9–12 mo)	
29	~90% (9–12 mo)	

Abbreviations: mo, months old; RCV, rubella-containing vaccine; SIA, supplemental immunization activity; yo, years old.

^a^The first 10 years of low vaccination coverage represent an example of RCV administered in the private-sector only [19]. Increased routine vaccination at 10 and 20 years represents introduction of RCV into national vaccine schedules and then expansion of the programs to capture more infants, respectively. SIAs at 10 and 18 years represent typical SIA efforts to vaccinate many young age groups.

**Table 2.** Assumed Data Error Assumptions Used to Infer Estimates of Proportion Immune (Vaccine Induced, Natural Infection Induced, and Total) in Figure 2^a^

Time Point	Vaccination Coverage Data Error Assumptions	Incidence Data Error Assumptions
6	Unavailable	Underreported by 95%
11	Overreported ~10%	Underreported by 92%
16	Overreported ~10%	Underreported by 88%
26	Overreported ~10%	Underreported by 85%

^a^We assume vaccination coverage, when available, is biased upwards [11] conservatively by ~10% [12], and reporting starts relatively low, improving as vaccine coverage improves. Given biased coverage estimates, knowledge of the age targets, timing of SIAs, and age-specific vaccine effectiveness, the age profile of vaccine-induced immunity, $\hat{v(a)}$ was reconstructed. Given biased incidence data, the proportion immune by natural infection, $\hat{r(a)}$, was estimated adjusting for underreporting (assuming 85% underreporting). These 2 (biased) estimates allow estimation of total proportion immune, $\hat{p(a)}$, by age assuming independence between the 2 sources of immunity, ie, $\hat{p\left(a\right)}=1-\left(\left(1-\hat{r\left(a\right)}\right)\left(1-\hat{v(a)}\right)\right)$.

#### Age-Specific Serological Profiles in Endemic Settings

In settings where vaccination coverage is low or absent, the age-specific serological profile is largely dominated by natural immunity (Figure 1A). The rate of acquisition of immunity with age (after loss of maternal immunity) is determined by the rate of transmission in the population. If transmission is low, acquisition of infection and thus immunity is slow. Susceptible individuals may not encounter an infected individual until adolescence or older. R_0_, the basic reproductive number representing the number of new infections per infectious individual in a completely susceptible population, is a commonly used measure of transmission potential that informs expected age-specific patterns of seropositivity [1]. Data detailing changes in the number of cases over time [10] or their distribution across ages [18] can be combined with mathematical models to estimate R_0_ and infer expected age-specific immunity patterns. In addition, the profile of cases accumulated across age strata may be used to reflect the cumulative proportion of immune individuals by age, although biases may emerge due to age-specific sensitivity of reporting.

Insensitivity of disease surveillance data can affect estimation of immunity gaps. Assuming optimistically that 5%–15% of cases are reported across a spectrum from endemicity to elimination (Table 2), Figure 2A illustrates how using case data to estimate the fraction of the population naturally immune over age, *r(a*) (Figure 2A2), results in biased estimates of the proportion seropositive by age, *p(a*) (Figure 2A3), in an endemic setting. Poor quality of reported vaccination coverage further complicates interpretation of case data for understanding age profiles of immunity. Countries without national rubella vaccination programs often have low levels of private healthcare sector vaccination [19] (Table 1). If these estimates are unreported, they could cause bias estimates related to measures of transmission (eg, R_0_) and age-specific seroprevalence profiles (Figures 2A1 and 2A3); see Supplementary Materials S2 for an empirical example.

## RESULTS

### Age-Specific Serological Profiles in Settings With Increased Vaccine Coverage

In areas where vaccination programs are well established, vaccine-derived immunity dominates the age-specific serological profile at younger ages. Older cohorts may have acquired natural immunity before high vaccination coverage was achieved, and maternal and natural immunity will continue to play some role.

Different strategies for vaccine administration (ie, routine vaccination or SIAs) mean that the history of vaccine delivery can result in distinct age-specific seroprevalence profiles (Figure 1B–D). In low-income countries, routine MCV was designed to reach children during the first year of life. More recently, children have access to a second dose of MCV, commonly in the second year of life [8]. Supplemental immunization activities are conducted periodically (usually every 2–5 years) over brief timeframes (usually days/weeks, although potentially months/years in large countries) at the national or subnational level and target specific age groups (eg, 9 months–5 years of age). Outbreak response campaigns also occur. Information on the history of routine and SIAs allow inference into the age profile of vaccine-derived immunity, but data on vaccination coverage are often inaccurate [7, 11, 12]. Without individual vaccine histories, combining SIA coverage [20], outbreak response campaigns [21], and (where they exist) routine second dose programs may overestimate susceptibility reduction, because vaccination may be disproportionately delivered to immune individuals. For example, one study estimated that 31% of eligible populations were never accessible by routine or campaign vaccination in Sierra Leone [12]. Even if vaccine coverage areas were known precisely, everyone does not develop protective immunity after vaccination [2], and vaccine-induced antibody levels may wane below the threshold for seropositivity [3], resulting in discrepancies between vaccination history and age-specific seroprevalence.

Uncertainties in vaccination coverage data combined with underreported incidence (Table 2) result in errors in estimates of the proportion seropositive by age (Figure 2B–D). Estimated vaccination coverage is likely to overestimate the proportion immunized, *v(a*) (Figure 2B1, 2C1, and 2D1), even after accounting for vaccine failure. Incomplete surveillance biases estimates of the proportion immune from natural infection, *r(a*) (Figure 2B2, 2C2, and 2D2), can further bias inferred seropositivity, *p(a*) (Figure 2B3, 2C3, and 2D3). By improving case surveillance, managers can reduce the degree of bias (Figure 2).

Immunity derived from natural infection remains a source of seropositivity in expanded control settings, but it is an erratic, potentially misleading one. For example, extended periods of low incidence after vaccine introduction may allow accumulation of susceptible individuals [1]. Eventually, their proportion may grow sufficiently large to sustain an outbreak. Thus, incidence provides a poor indicator of population immune status: low case numbers can reflect either sustained, high levels of population immunity or an increasing risk of an outbreak. Imperfect surveillance combined with uncertainty in vaccination coverage data further complicate estimates of age-specific population immunity.

#### Age-Specific Serological Profiles in Near-Elimination Settings

**Figure 3.**
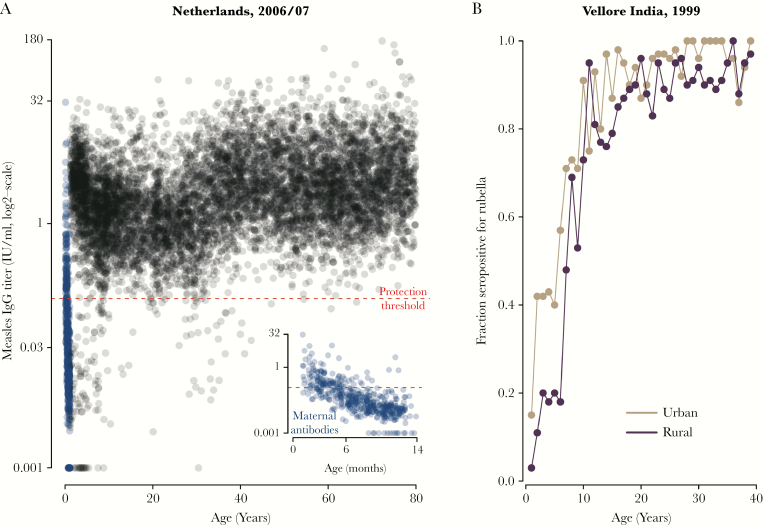
Example serological survey data. (A) Measles immunoglobulin G titers for a representative sample of the Netherlands [23]; infants with maternally derived antibodies in blue (main plot and inset); threshold for protection (immunity) shown by the horizontal dashed line. By contrast with categorical data (positive versus negative, see previous figures), this quantitative measure reveals the distribution of antibody concentrations relative to the threshold of protection, and it can be used to study maternal antibody decay (see main text). (B) Fraction of individuals seropositive from rubella antibodies in urban and rural Vellore, India [40]. Associated estimates of the magnitude of transmission can be combined with age fertility to infer the burden of congenital rubella syndrome (CRS) prevaccination. For this example, assuming that age-specific contact patterns and private-sector vaccination coverage were comparable in both settings, these patterns suggest higher transmission in urban than rural settings, because the increase in age-specific seroprevalence is faster in urban settings. Accounting for differences in fertility, higher transmission of rubella in urban Vellore contributed to lower estimated risk of CRS compared with rural Vellore [40].

In near-elimination settings, the prevalence of immunity derived from natural infection decreases and vaccine-derived immunity increases. Reconstructing immunity profiles without serology is complicated by vaccination coverage uncertainty, absent or rare case data from surveillance, and the resultant decline in positive predictive value of cases reported [22], unless all cases are laboratory-confirmed. Estimating the age-specific immunological profile is an important factor in identifying vaccination age targets to achieve and sustain elimination. One particular near-elimination issue is maternally derived immunity. Given that maternal antibodies neutralize vaccine virus, vaccine efficacy increases with age as maternal antibodies wane (Figure 3A). Accordingly, administration of the first dose of MCV is usually delayed until 9 or 12–15 months of age to ensure that infants are free of maternal antibodies. However, vaccinated mothers transfer a lower level of measles virus-specific antibodies to their children than naturally infected mothers [23], potentially leaving these children susceptible to measles at an earlier age and raising questions about shifting the age of vaccination younger (see below). The ability to robustly characterize such nuanced patterns without serology is challenging.

### Addressing Key Questions for the Control and Elimination of Measles and Rubella

#### Strategizing Effective Targeting of Vaccination to Control and Eliminate Infection

In mature vaccination programs, additional efforts would ideally target immunity gaps that could allow outbreaks [8]. Populations are at risk of an outbreak when the number (density) of susceptible individuals is sufficiently large. Susceptible individuals are distributed over age (and space; see Supplementary Materials S3). Age-specific seroprevalence estimates, combined with data on mixing-patterns by age and vaccination coverage, can help estimate susceptibility and characterize outbreak risk [7, 24], thus helping program managers prioritize strategies to close immunity gaps.

One way to tackle immunity gaps is by applying modifications to the timing and targeting of vaccination campaigns. For example, low seropositivity in age classes thought to be important for transmission (eg, school children) suggests a need to increase vaccination coverage in this age group (assuming that a sensitive laboratory assay and the appropriate seropositivity cutoff have been used [25], because antibody levels wane after vaccination, especially in the absence of boosting from exposure to wild-type virus [15, 16]). For example, observing low immunity in children aged 7–10 years (Figure 1B) before a planned vaccination campaign strongly supports extending the age range of the campaign beyond age 5 years to close this immunity gap.

Serological data could also be used to help determine the need for a vaccination campaign. Serosurveillance is conducted in high-income countries such as Japan [26], Australia [27], the United Kingdom, and the Netherlands [28], and it has contributed to vaccine policy recommendations such as initiating catch-up campaigns [29, 30]. A serosurveillance system that triggers vaccination campaigns may be particularly valuable in near-elimination settings because sparse case data are a poor indicator of increases in population susceptibility, and immunity gaps may go undetected until an outbreak occurs [31]. Historically, these outbreaks have had age distributions that deviate from expected patterns of age susceptibility based on historical vaccination coverage data (eg, Malawi [21]). Serosurveys can reveal changes in the age distribution of immunity, providing an opportunity to conduct campaigns to fill immunity gaps before outbreaks occur [32]. More importantly, survey results must be available promptly, so that vaccination campaigns can be planned and implemented rapidly in response to serological data.

One additional benefit of age-specific seroprevalence is the potential to fine-tune age of routine vaccination in high-vaccine settings. The ideal age of the first dose of MCV will optimize vaccine effectiveness without increasing the number or severity of measles cases [33]. Although the WHO Americas Region successfully sustained measles elimination after an increase in the age for the first dose of measles vaccine [6], in other regions cases before the age of 9 months remain a concern. Because mothers with vaccine-induced immunity have lower antibody levels than those with infection-induced immunity, babies born to the former have lower levels of maternal antibody and become susceptible at an earlier age. Therefore, the transition to elimination could result in more unprotected infants below the age of routine vaccination [23]. In the WHO African region, high birth rates mean that this could eventually translate to large numbers of susceptible individuals capable of maintaining measles virus transmission [34], an effect potentially amplified by high rates of human immunodeficiency virus (HIV) infection, which reduces the efficiency of transplacental transfer of maternal measles-specific antibodies [35]. Because more countries have extended periods of low measles incidence and more women of child-bearing age have vaccine-induced immunity, the optimal age for routine administration of first and second doses of MCV becomes an important policy question that serology could potentially inform. However, given the narrow age window affected, the programmatic scale of the interventions implied, and the scope of impact of any change, detailed data beyond cross-sectional, age serology (eg, clinical trials) may be required to inform policy decisions.

#### Evaluating the Effectiveness of Vaccination Programs Against Infections

Serology can be used to assess the effectiveness of vaccination activities, the impact on disease burden, and the progress towards elimination.

The role of SIAs in achieving measles elimination is receiving increasing scrutiny because of the high costs and human resource needs required to successfully conduct campaigns. Serosurveys have been suggested as one option to evaluate their success [17]. However, cross-sectional serosurveys cannot currently distinguish immunity from natural infection from vaccine-induced immunity (Figure 2), which complicates the use of a single postcampaign serosurvey to evaluate SIA impact (although detection of IgM antibodies within weeks of an SIA may indicate immunization of a susceptible individual, because individuals with prior immunity should not mount an IgM response). Conducting pre- and postcampaign serological surveys does allow managers to measure the increase in immunity due to SIAs, as demonstrated in England and Wales [30], Australia [36], and research settings in Ethiopia [37] and Kenya [38].

If a precampaign serosurvey is not feasible, serosurveys can determine whether target immunity prevalence has been reached, without drawing specific conclusions about the vaccination campaign. Otherwise, nuanced inferential tools combining data from serosurveys with age-specific disease incidence and the history of vaccination can estimate vaccination campaign effectiveness, although uncertainties with these data sources (see above) remain a limitation.

Age-specific serological data are also a valuable resource to indirectly infer disease burden. The burden of congenital rubella syndrome (CRS) is difficult to measure directly, given the complexity of diagnosis and difficulty of reporting in settings with limited medical resources [39]. Estimates obtained by combining the (1) age-specific force of infection derived from age-specific serological data with the (2) age profile of fertility and risk during pregnancy provide the only estimate of CRS in many countries [40] (see Figure 3B).

Finally, serological surveys provide an additional source of population immunity estimates, a necessary line of evidence for measuring progress towards elimination and verifying elimination [41, 42]. The WHO Americas Region success in eliminating measles and rubella largely without reliance on serological data to inform vaccination policy is likely to have been a result of the robustness and consistency of their vaccination programs. Other WHO regions may require broader data streams to describe their unique transmission dynamics. In African countries, vaccination coverage varies substantially within and between countries [43], and data on SIA coverage are suboptimal, especially regarding their effectiveness in reaching previously unimmunized persons [12]. Vaccine refusal in European countries, and increasingly elsewhere, can result in patches of susceptible individuals associated with considerable outbreak risk, given high importation rates of infected individuals due to inter- and intramigrations [44]. Seroprevalence profiles are correspondingly hard to anticipate [7, 21].

## DISCUSSION

### Serological Survey Challenges

High-quality, cross-sectional household serological surveys with blood specimen collection require the following: (1) substantial financial resources and time commitment, (2) logistical capacity and skilled personnel to design and conduct a serosurvey that is generalizable to the target population, (3) laboratories and laboratory expertise to perform serological assays with quality control and assurance, and (4) expertise in statistical analysis to interpret serological data [17]. Thus, serosurveys are typically not prioritized in low- or lower-middle income countries, but there is potential to make them more feasible and sustainable by the following: (1) expanding capacities in conducting high-quality household surveys such as vaccination coverage surveys [11]; (2) including serology in vaccination coverage and/or multipurpose household surveys (as already is done for HIV [45]); (3) expanding existing sentinel site surveillance systems to include measles/rubella serology; (4) expanding the scope of serological surveillance to other vaccine-preventable and emerging infectious diseases (eg, multiplex assays); and (5) standardizing specimen collection, testing, and interpretation of serological results (see Supplementary Materials S4 for further discussion).

The WHO is rolling out updated guidance for the conduct of high-quality vaccination coverage surveys [46]. Established household survey programs such as the Demographic and Health Surveys offer opportunities for inclusion of serology in many countries. However, major barriers remain, including the following: difficulty in ensuring high participation rates, especially for invasive samples (eg, blood, which often has the benefit of reduced laboratory uncertainty) [47]; difficulty in standardizing different laboratory assays [47, 48]; and challenges in defining appropriate cutoffs for seropositivity [48] (although expanded deployment of statistical methods such as mixture models could formally address individual variability in cutoffs [49]).

For measles, a cutoff of 120 IU/L is proposed as indicating protection from infection, from plaque reduction neutralization assay results on blood samples obtained before and after a measles outbreak in American college students [50]. This cutoff may not be appropriate (1) if less sensitive, nonfunctional assays such as enzyme immunoassays (EIAs) are used [47] and (2) when seeking evidence of past immunization (in which case a lower threshold may be appropriate), irrespective of whether antibody levels have persisted above the putative fully protective threshold. Likewise, for rubella, antibody levels wane after vaccination and EIAs lead to a high number of false-negative results [48]. Careful examination of the data can lead to a more appropriate cutoff choice, aligned to the survey objectives, rather than using universal cutoffs (eg, 120 IU/L for measles; 10–15 IU/mL for rubella). Much remains to be done to identify, or develop, field-friendly assays that consistently provide readily interpretable data from surveys in low- and middle-income countries [17, 25].

## CONCLUSIONS

As countries approach measles and rubella elimination goals, the age profile of immunity and the relative contribution of natural and vaccine-derived immunity change. High-quality serosurveys allow explicit characterization of the distribution of immunity at a particular time point, but they also provide mangers with an opportunity to evaluate assumptions made about natural transmission dynamics and vaccine program performance. However, given the costs and challenges inherent in deploying serological surveys, the potential gain in inference should be considered carefully before such surveys are planned. Dynamic models can link serology, clinical, and programmatic surveillance to generate robust estimates of the profile of immunity. Immunity gaps identified through multiple data sources, including serology, can be used to target specific interventions and improve routine programs to prevent future gaps. Finally, investment in serosurveillance for the goal of measles and rubella surveillance could form the foundation of broader serosurveillance efforts as multiplex assays become increasingly available.

## Supplementary Materials

Supplementary materials are available at *The Journal of Infectious Diseases* online. Consisting of data, methods, and discussion provided by the authors to benefit the reader, the posted materials are not copyedited and are the sole responsibility of the authors, so questions or comments should be addressed to the corresponding author.

Supplementary Information

## Notes

***Disclaimer.*** The funding source played no role in the design of this paper.

***Financiap support.*** This study was funded by the Bill & Melinda Gates Foundation.

***Potential conflicts of interest.*** W. J. M., J. L., M. J. F., C. J. E. M., and K. H. report grants from the Bill & Melinda Gates Foundation, outside the submitted work. F. T. C. reports that she is a consultant for the Bill & Melinda Gates Foundation. All authors have submitted the ICMJE Form for Disclosure of Potential Conflicts of Interest. Conflicts that the editors consider relevant to the content of the manuscript have been disclosed.

## References

[1] Gay NJ The theory of measles elimination: implications for the design of elimination strategies. J Infect Dis 2004; 189(Suppl 1):S27–35. 15106086 10.1086/381592

[2] Pannuti CS, Morello RJ, de Moraes JC, et al Identification of primary and secondary measles vaccine failures by measurement of immunoglobulin G avidity in measles cases during the 1997 Sao Paulo epidemic. Clin Diagn Lab Immunol 2004; 11:119–22. 14715557 10.1128/CDLI.11.1.119-122.2004 PMC321355

[3] de Melker H, Pebody RG, Edmunds WJ, et al The seroepidemiology of measles in Western Europe. Epidemiol Infect 2001; 126:249–59. 11349976 10.1017/s0950268801005234 PMC2869690

[4] Dabbagh A, Patel MK, Dumolard L, et al Progress toward regional measles elimination—worldwide, 2000–2016. MMWR Morb Mortal Wkly Rep 2017; 66:1148–53. 29073125 10.15585/mmwr.mm6642a6 PMC5689104

[5] World Health Organization. Global Measles and Rubella: Strategic Plan 2012–2020, 2012 http://apps.who.int/iris/bitstream/handle/10665/44855/9789241503396_eng.pdf;jsessionid=4FC1AD7464183C1904A0993EC10E0555?sequence=1. Accessed 2 April 2018.

[6] Andrus JK, de Quadros CA, Solórzano CC, Periago MR, Henderson DA Measles and rubella eradication in the Americas. Vaccine 2011; 29(Suppl 4):D91–6. 22185837 10.1016/j.vaccine.2011.04.059

[7] Trentini F, Poletti P, Merler S, Melegaro A Measles immunity gaps and the progress towards elimination: a multi-country modelling analysis. Lancet Infect Dis 2017; 17:1089–97. 28807627 10.1016/S1473-3099(17)30421-8

[8] World Health Organization. Measles vaccines: WHO position paper. Wkly Epidemiol Rec 2017; 92:205–28.28459148

[9] Takahashi S, Metcalf CJ, Ferrari MJ, et al Reduced vaccination and the risk of measles and other childhood infections post-Ebola. Science 2015; 347:1240–2. 25766232 10.1126/science.aaa3438 PMC4691345

[10] Bjornstad ON, Finkenstadt BF, Grenfell BT Dynamics of measles epidemics: estimating scaling of transmission rates using a time series SIR model. Ecol Monogr 2002; 72:169–84.

[11] Cutts FT, Izurieta HS, Rhoda DA Measuring coverage in MNCH: design, implementation, and interpretation challenges associated with tracking vaccination coverage using household surveys. PLoS Med 2013; 10:e1001404. 23667334 10.1371/journal.pmed.1001404 PMC3646208

[12] Lessler J, Metcalf CJ, Grais RF, Luquero FJ, Cummings DA, Grenfell BT Measuring the performance of vaccination programs using cross-sectional surveys: a likelihood framework and retrospective analysis. PLoS Med 2011; 8:e1001110. 22039353 10.1371/journal.pmed.1001110 PMC3201935

[13] World Health Organization. Measles and rubella surveillance data Available at: http://www.who.int/immunization/monitoring_surveillance/burden/vpd/surveillance_type/active/measles_monthlydata/en/. Accessed 12 Febuary 2018.

[14] World Health Organization. Rubella vaccines: WHO position paper. Wkly Epidemiol Rec 2011; 86:301–16. 21766537

[15] Moss WJ, Scott S The immunological basis for immunization series: module 7: measles—Update 2009. In: World Health Organization, ed. Immunization Vaccines and Biologicals, World Health Organization; 2009 http://www.who.int/immunization/documents/ISBN9789241597555/en/. Accessed 2 April 2018.

[16] Best JM, Reef S Immunological basis for immunization: module 11: rubella. In: World Health Organization, ed. Immunization Vaccines and Biologicals, World Health Organization; 2008 http://www.who.int/immunization/documents/ISBN9789241596848/en/. Accessed 2 April 2018.

[17] Cutts FT, Hanson M Seroepidemiology: an underused tool for designing and monitoring vaccination programmes in low- and middle-income countries. Trop Med Int Health 2016; 21:1086–98. 27300255 10.1111/tmi.12737

[18] Lessler J, Metcalf CJ Balancing evidence and uncertainty when considering rubella vaccine introduction. PLoS One 2013; 8:e67639. 23861777 10.1371/journal.pone.0067639 PMC3702572

[19] Robertson SE, Cutts FT, Samuel R, Diaz-Ortega JL Control of rubella and congenital rubella syndrome (CRS) in developing countries, Part 2: Vaccination against rubella. Bull World Health Organ 1997; 75:69–80. 9141752 PMC2486979

[20] Li S, Ma C, Hao L, et al Demographic transition and the dynamics of measles in six provinces in China: a modeling study. PLoS Med 2017; 14:e1002255. 28376084 10.1371/journal.pmed.1002255 PMC5380361

[21] Minetti A, Kagoli M, Katsulukuta A, et al Lessons and challenges for measles control from unexpected large outbreak, Malawi. Emerg Infect Dis 2013; 19:202–9. 23343504 10.3201/eid1902.120301 PMC3559033

[22] Cutts FT, Brown DW The contribution of field tests to measles surveillance and control: a review of available methods. Rev Med Virol 1995; 5:35–40.

[23] Waaijenborg S, Hahné SJ, Mollema L, et al Waning of maternal antibodies against measles, mumps, rubella, and varicella in communities with contrasting vaccination coverage. J Infect Dis 2013; 208:10–6. 23661802 10.1093/infdis/jit143 PMC4043230

[24] Abrams S, Kourkouni E, Sabbe M, Beutels P, Hens N Inferring rubella outbreak risk from seroprevalence data in Belgium. Vaccine 2016; 34:6187–92. 27840011 10.1016/j.vaccine.2016.10.072

[25] Dimech W, Mulders MN A review of testing used in seroprevalence studies on measles and rubella. Vaccine 2016; 34:4119–22. 27340096 10.1016/j.vaccine.2016.06.006

[26] Infectious Disease Surveillance Center, National Institute of Infectious Diseases, National Epidemiological Surveillance of Vaccine-Preventable Diseases (NESVPD). Ministry of Health Labour and Welfare(MHLW). Committee of NESVPD in National Institute of Infectious Diseases: Procedure for the National Epidemiological Surveillance of Vaccine-Preventable Diseases; 2005–2015. http://idsc.nih.go.jp/yosoku/index-E.html. Accessed 2 April 2018.

[27] Gidding H Australia’s national serosurveillance program. N S W Public Health Bull 2003; 14:90–3. 12806408 10.1071/nb03027

[28] Osborne K, Weinberg J, Miller E The European Sero-Epidemiology Network (ESEN). Euro Surveill 1997; 2:29–31. https://www.ncbi.nlm.nih.gov/pubmed/12631820. 12631820 10.2807/esm.02.04.00167-en

[29] Gay NJ, Hesketh LM, Morgan-Capner P, Miller E Interpretation of serological surveillance data for measles using mathematical models: implications for vaccine strategy. Epidemiol Infect 1995; 115:139–56. 7641827 10.1017/s0950268800058209 PMC2271572

[30] Gay N, Ramsay M, Cohen B, et al The epidemiology of measles in England and Wales since the 1994 vaccination campaign. Commun Dis Rep CDR Rev 1997; 7:R17–21. 9046124

[31] Goodson JL, Masresha BG, Wannemuehler K, Uzicanin A, Cochi S Changing epidemiology of measles in Africa. J Infect Dis 2011; 204(Suppl 1):S205–14. 21666163 10.1093/infdis/jir129

[32] Lessler J, Metcalf CJ, Cutts FT, Grenfell BT Impact on epidemic measles of vaccination campaigns triggered by disease outbreaks or serosurveys: a modeling study. PLoS Med 2016; 13:e1002144. 27727285 10.1371/journal.pmed.1002144 PMC5058560

[33] Metcalf CJ, Klepac P, Ferrari M, Grais RF, Djibo A, Grenfell BT Modelling the first dose of measles vaccination: the role of maternal immunity, demographic factors, and delivery systems. Epidemiol Infect 2011; 139:265–74. 20525415 10.1017/S0950268810001329 PMC4756473

[34] McKee A, Ferrari MJ, Shea K The effects of maternal immunity and age structure on population immunity to measles. Theor Ecol 2015; 8:261–71. 26140058 10.1007/s12080-014-0250-8 PMC4485449

[35] Scott S, Moss WJ, Cousens S, et al The influence of HIV-1 exposure and infection on levels of passively acquired antibodies to measles virus in Zambian infants. Clin Infect Dis 2007; 45:1417–24. 17990222 10.1086/522989

[36] Gilbert GL, Escott RG, Gidding HF, et al Impact of the Australian Measles Control Campaign on immunity to measles and rubella. Epidemiol Infect 2001; 127:297–303. 11693507 10.1017/s0950268801005830 PMC2869749

[37] Nigatu W, Samuel D, Cohen B, et al Evaluation of a measles vaccine campaign in Ethiopia using oral-fluid antibody surveys. Vaccine 2008; 26:4769–74. 18644417 10.1016/j.vaccine.2008.07.005

[38] Ohuma EO, Okiro EA, Bett A, et al Evaluation of a measles vaccine campaign by oral-fluid surveys in a rural Kenyan district: interpretation of antibody prevalence data using mixture models. Epidemiol Infect 2009; 137:227–33. 18544176 10.1017/S0950268808000848 PMC2696684

[39] Lawn JE, Reef S, Baffoe-Bonnie B, Adadevoh S, Caul EO, Griffin GE Unseen blindness, unheard deafness, and unrecorded death and disability: congenital rubella in Kumasi, Ghana. Am J Public Health 2000; 90:1555–61. 11029988 10.2105/ajph.90.10.1555 PMC1446363

[40] Vynnycky E, Adams EJ, Cutts FT, et al Using seroprevalence and immunisation coverage data to estimate the global burden of congenital rubella syndrome, 1996–2010: a systematic review. PLoS One 2016; 11:e0149160. 26962867 10.1371/journal.pone.0149160 PMC4786291

[41] World Health Organization. Framework for verifying elimination of measles and rubella. Wkly Epidemiol Rec 2013; 88:89–99. 23540051

[42] World Health Organization. Guidelines of Verification of Measles Elimiation in the Western Pacific Region, World Health Organization; 2013. http://www.wpro.who.int/immunization/documents/measles_elimination_verification_guidelines_2013/en/. Accessed 2 April 2018.

[43] Takahashi S, Metcalf CJ, Ferrari MJ, Tatem AJ, Lessler J The geography of measles vaccination in the African Great Lakes region. Nat Commun 2017; 8:15585. 28541287 10.1038/ncomms15585 PMC5458501

[44] World Health Organization. Surveillance Guidelines for Measles, Rubella and Congenital Rubella Syndrome in the WHO European Region In: Regional Office for Europe, ed. World Health Organization; 2012. http://www.who.int/immunization/documents/measles_rubella_eur_08_5082738/en/. Accessed 2 April 2018. 23762964

[45] Fishel JD, Garrett D. Performance of Enzyme Immunoassays for HIV Serology in Surveys Donducted by the Demographic and Health Surveys Program. DHS Comparative Reports No 39. Rockville, Maryland: ICF International, 2016.

[46] World Health Organization. Vaccination Coverage Cluster Survey: Reference Manual, Version 3 Working Draft (updated July 2015): Immunization Vaccines and Biologicals, World Health Organization; 2015. http://www.who.int/entity/immunization/monitoring_surveillance/Vaccination_coverage_cluster_survey_with_annexes.pdf?ua=1. Accessed 2 April 2018.

[47] Mulders MN, Rota PA, Icenogle JP, et al Global measles and rubella laboratory network support for elimination goals, 2010–2015. MMWR Morb Mortal Wkly Rep 2016; 65:438–42. 27148917 10.15585/mmwr.mm6517a3

[48] Huzly D, Hanselmann I, Neumann-Haefelin D, Panning M Performance of 14 rubella IgG immunoassays on samples with low positive or negative haemagglutination inhibition results. J Clin Virol 2016; 74:13–8. 26638144 10.1016/j.jcv.2015.11.022

[49] Hens N, Shkedy Z, Aerts M, Faes C, Van Damme P, Beutels P Statistics for biology and health. In: Modeling Infectious Disease Parameters Based on Serological and Social Contact Data: A Modern Statistical Perspective. Statistics for biology and health. New York: Springer, 2012.

[50] Chen RT, Markowitz LE, Albrecht P, et al Measles antibody: reevaluation of protective titers. J Infect Dis 1990; 162:1036–42. 2230231 10.1093/infdis/162.5.1036

