# Supplementary material for: Benefits and Challenges in Using Seroprevalence Data to Inform Models for Measles and Rubella Elimination
Source: J Infect Dis. 2018 Mar 19;218(3):355–64. doi: 10.1093/infdis/jiy137 (PMC6049004; doi:10.1093/infdis/jiy137)
Supplement: Supplementary Information [file jiy137_suppl_supplementary_information.docx]

**Supplements to**: **Benefits and challenges in using sero-prevalence data to inform models for measles and rubella elimination; Winter et al.**

**Supplement S1: Methods**

***Methods to Create Figure 1.*** In order to delineate expectation for age-specific immunity profiles, we used an age-structured discrete-time deterministic mathematical model to simulate rubella dynamics over 30 years from an endemic setting with low vaccination to elimination setting with high vaccination. Figure 1 displays the results of this simulated example of rubella dynamics. The following three sections discuss the details of the model structure and parameterization, and the model output used to create Figure 1.

*Age-Structured MSIRV Transmission Model*

To simulate rubella transmission dynamics, we used an age-structured discrete time stochastic transmission model that incorporates both epidemiological and demographic transitions, building on framework introduced by [1, 2]. The model structure described here was originally presented in [3] and was used to describe rubella dynamics in [4]. We structured the population into five epidemiological stages (maternally immune ‘M', susceptible ‘S', infected ‘I', recovered ‘R', and vaccinated ‘V', taken to indicate the effectively vaccinated), and 321 age classes (240 monthly strata from ages 1 month through 19 years, and 81 yearly strata from ages 20 to 100 years). The key feature of the model is a matrix that at every time-step defines transitions from every possible epidemiological stage and age class combination to every other possible epidemiological stage and age class combination.

We take a two-step approach in order to describe the large transition matrix. Step one is to define epidemiological transitions, ignoring demographic transitions (aging and survival). Matrix **A_a,t_**, captures transitions between each epidemiological stage within each age class *a* and discrete time-step *t*, where the time-step *t* was set to the approximate generation time of rubella at slightly greater than 2 weeks (i.e., we assume 24 infection generations per year). Given that rubella has a generation time of about 18 days, our model is conservative by slightly overestimating yearly transmission rates; however, sensitivity analyses suggest the effect of vaccination on burden estimates are robust to this parameter [4]. Matrix **A_a,t_** is defined as,


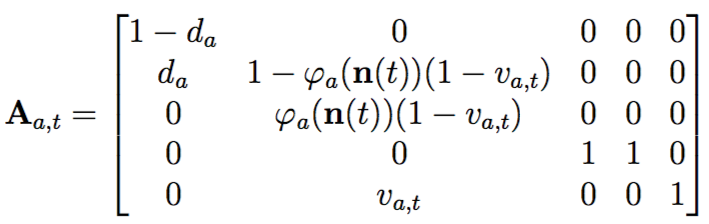


The five rows from left to right and columns from top to bottom represent the ‘M', ‘S', ‘I', ‘R', and ‘V' epidemiological stages, respectively. In the transition matrix, d_a_ is the probability of losing maternal immunity by age class *a*, $\varphi_{a}$ is the probability an individual in age class *a* becomes infected, and v_a,t_ is the probability an individual in age class *a* and time-step *t* is successfully vaccinated. The probability of infection by age, $\varphi_{a}$ (also called the age-specific force of infection, FOI) is a function of **n**(t), a vector describing the population at time *t*, defined as,

$$\mathbf{n}\left( t \right)={(M_{1,t}, S_{1,t}, I_{1,t}, R_{1,t}, V_{1,t}, M_{2,t}, \ldots V_{z,t})}^{T}$$

according to

$$\varphi_{a}(\mathbf{n}(t)) = 1- exp (\frac{-\sum_{j} \beta_{a,j,t} I_{j,t}^{\gamma}}{\sum\mathbf{n}(t)})$$

where *z* is the total number of age classes (here *z* = 321), $\beta_{a,j,t}$ is the rate of transmission between individuals in age class *a* and *j* at time-step *t*, also known as the Who-Acquires-Infection-From-Whom (WAIFW) matrix, and $I_{j,t}^{\gamma}$ is the number of infected individuals in age class *j* and time-step *t*, while $\gamma$ captures the non-modeled heterogeneities in age mixing [5, 6] and the effects of discretization of the underlying continuous process [7]. We fix $\gamma$ at 0.97 reflecting values obtained for measles in England and Wales [5], because discrete-time models that do not incorporate this exponent result in unrealistically unstable dynamics prone to frequent extinction. Given that rubella transmission is frequency dependent, we divide the number of infected individuals in each age class by the total population size at time-step t ($\sum\mathbf{n}(t)$).

The second step is to define the full transition matrix, **A**(**n**(*t*)), that includes both epidemiological transitions captured in matrix **A**_a,t_, and demographic transitions (aging and survival). This matrix is used to project the entire population forwards via aging, mortality, and transmission dynamics and is defined as,


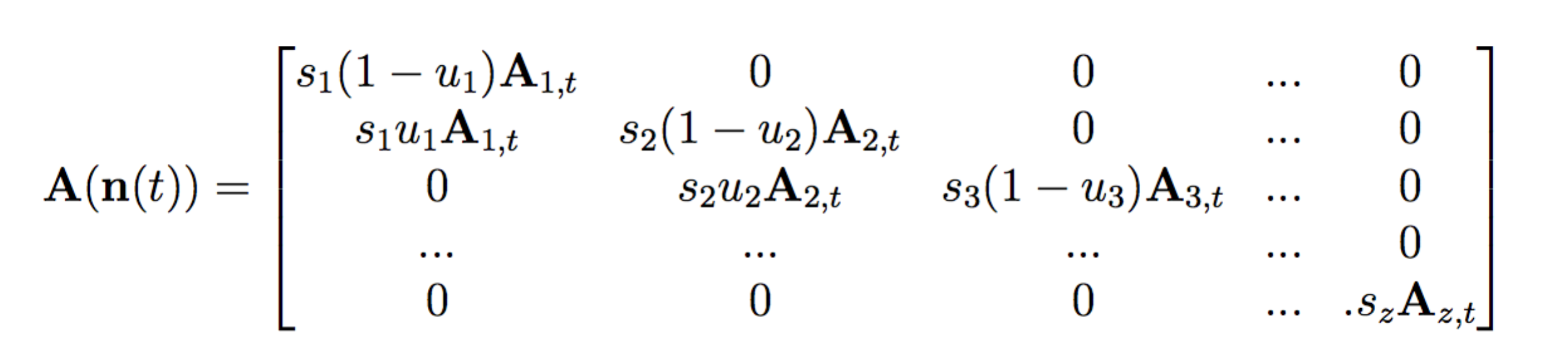


where s_a_ is the probability that an individual in age class *a* survives, u_a_ is the rate of aging out of age class *a*, and **A_1,t_** , **A_2,t_**, etc., as defined above for **A_a,t_**. The dynamics of the whole population are then projected forward in time, such that

**n**(*t* + 1) = **A**(**n**(*t*))**n**(*t*) + **B_t_**

where **B_t_** is a vector representing the number of births at time *t*, defined as,

**B_t_** = (*b_t_,* 0*,* 0*, ...*0)^T^

*Parameterizing the Model*

We combine broadly known rubella epidemiology with realistic demographic rates in order to simulate rubella dynamics. The following sections describe our parameterization of epidemiological and demographic features of this model.

Epidemiological Parameters

I. Transmission across ages and over time, $\beta_{a,j,t}$

Transmission to individuals in age class *a*, from individuals in age class *j* for each time-step *t* is defined by $\beta_{a,j,t}=\bar{\beta_{a,j,}}(1+\alpha cos(2\pi t))$, where $\bar{\beta_{a,j}}$ is mean transmission from individuals in age class *j* to age class *a*, and $\alpha$ is a parameter controlling the magnitude of seasonal fluctuations.

1. Seasonal forcing, $\alpha$

The model accounts for seasonal forcing [8-10], where intensity of transmission varied over each year (affecting all age groups the same). Previous validation of this model has shown that model results for the burden of CRS were robust to the magnitude of seasonal fluctuations [4]; we set $\alpha$ to 0.40 and held it constant over time (Figure 1b).

1. Age-contact mixing

Mean transmission from individuals in age class *j* to age class *a,* $\bar{\beta_{a,j}}$, was estimated by rescaling population-adjusted age-contact rates to reflect the assumed basic reproductive number of rubella. Empirical estimates from large-scale qualitative studies generally converge on assortative age-contact patterns relevant for infections transmitted via the respiratory route, specifically among school ages [11-13]. Accordingly, in previous applications of this age-structured MSIRV model we assumed age-specific contact patterns estimated from those characterized in the European POLYMOD study based on diary entries [11].

c. Basic reproductive number of rubella, R_0_

We assigned the basic reproductive number (i.e., R_0_, defined as the average number of people a ‘typical' infected individual will infect in a fully susceptible population) of rubella to be 6. Estimates of R_0_ for rubella based on population or community-based serological data have ranged from 3.0 in European countries [14] to 11.8 in Addis Ababa, Ethiopia [15].An in-depth empirical analysis of age-structured rubella incidence data from 40 African countries, which estimated a median R_0_ was 5.2 (90% CI 4.0-6.7) [16].

II. Maternal immunity by age, d_a_

The duration of protection by rubella maternal antibodies ranges between 3 and 9 months [17, 18]; accordingly, we modeled the probability of remaining in the maternally immune epidemiological stage over age (1-d_a_) as an exponential decay function with a constant rate of 0.95 per month (Figure 1a).

III. Successful vaccination by age and time, v_a,t_

The probability of successful vaccination for individuals in age class *a* and time-step *t* (v_a,t_) is obtained by multiplying the probability of vaccination at age *a* and time-step *t,* by a vaccination efficacy rate over age *a*.

a. Probability of vaccination

The probability of vaccination is calculated as the cumulative probability that an individual had access to a vaccine at age *a* (via routine immunizations or campaign) scaled to reflect the assumed vaccination coverage for age *a* and time-step *t*. The age-specific probability of access to routine vaccination was extracted from empirical estimates for Zambian children from [19]. The probability of access to a campaign vaccination was assumed to be 0.95 across all ages in the target age range, given that vaccination campaign coverage is often overestimated, as it typically is derived from administrative coverage estimates [20]. Over the course of 30 years, we assumed routine vaccination coverage among children 12 months old was less than 10% for the first 10 years, between 48% and 52% years 11 to 20, and between 87% and 93% years 20 to 30. We additionally included vaccination campaigns (i.e., SIAs) targeting children ages 1 to 5 years old at 10 years with coverage of 70%, and 18 years with coverage of 80%.

b. Vaccine efficacy

Vaccination efficacy rate over age was empirically estimated from data extracted from [21]. We forced vaccine efficacy over age to saturate at 97%.


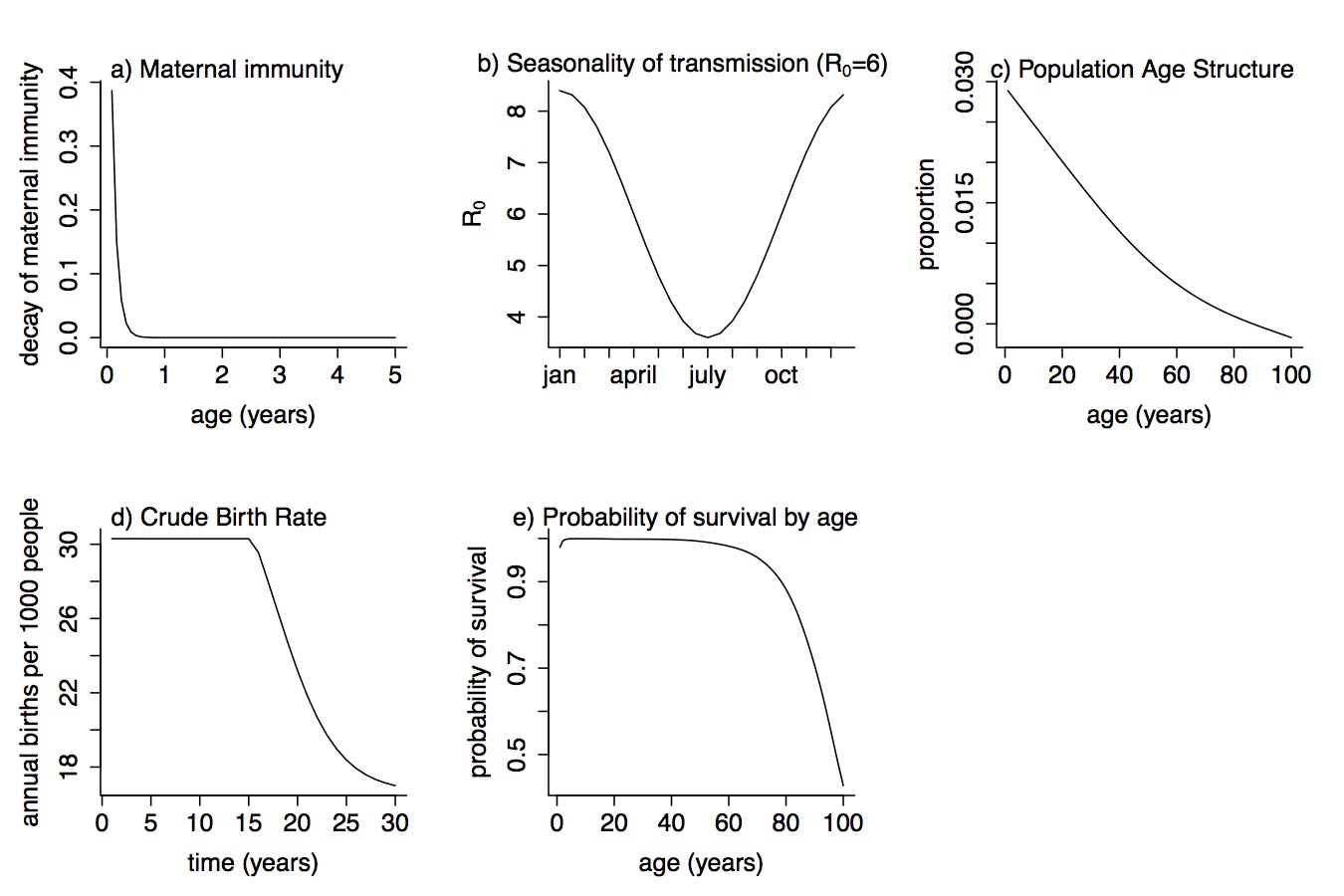


**Figure 1:** Parameter inputs: a) maternal immunity by age, b) seasonality of transmission over a year (assuming R_0_ is 6), c) starting population age structure, d) crude birth rate over time, e) age-specific probability of survival.

Demographic Parameters

The model requires the following demographic parameters: total population size, population age structure, crude birth rate over time, and age-specific survival rates.

I. Births over time, b_t_

The number of births per time-step *t* (b_t_) were estimated by multiplying the crude birth rate per time-step *t* (i.e., annual crude birth rate divided by 24 generations in a year) by the total population at time-step *t* ($\sum\mathbf{n}(t)$). The assumed annual crude birth rate over time is displayed in Figure 1d.

II. Deaths by age and over time, s_a_

Age-specific survival probabilities (s_a_), were held constant over time (Figure 1e).

III. Aging, u_a_

We assumed a constant rate of aging into the next age class (u_a_ = 1 / (length of age class *a* in years $\times$ 24)).

IV. Starting endemic population, **n**(*t=1*)

To simulate rubella dynamics, we first need a rubella endemic population (i.e., **n**(1)). We began with fully susceptible populations based on the age structure displayed in Figure 1c and population size of 1,000,000 (i.e., $\sum\mathbf{n}\left( t=1 \right)$). In order to move beyond the transient non-seasonal outbreaks to populations representing endemic rubella, we seed infected individuals into each population and iteratively simulated rubella dynamics for four 20-year increments assuming constant births and deaths. At the end of each 20 year cycle, we rescale the mean WAIFW ($\bar{\beta_{a,j}}$) by the assumed R_0_ and the starting population and age structure, and then simulate again for four times total. The result is populations representing endemic rubella (i.e., **n**(1)).

*Model Output*

At every time-step the model outputs the number of individuals in each epidemiological stage and age class. Therefore, we were easily able to sum the number of infected individuals age per time point *t* to get the time series of incident cases (top panel Figure 1). Additionally, at the beginning of simulated year 6, 11, 16, and 26 we extracted the age-specific proportion immune by summing the number of individuals in the “maternally immune,” “infected,” “recovered,” and “vaccinated” epidemiological stage per age and dividing by the total number of individuals per age (bottom panel Figure 1).

***Methods to Create Figure 2.*** In Figure 2, we compare the simulated results to estimates of proportion immune inferred from vaccination and incidence data. The inferred estimates of proportion vaccinated by age (i.e., $v(a)$) and proportion recovered from natural infection by age (i.e., $\hat{r(a)}$).) are based on assumptions of biases in these two data sources. These assumptions are then applied to the ‘true’ values of vaccination coverage (per routine or SIA coverage assumed for the simulation) and incident cases (per the simulation output).

We assumed that vaccination coverage estimates by age via routine vaccination and SIAs were unavailable at time point 6 and available but over-reported at time points 11, 16 and 26. The over-reporting error per age group was randomly drawn from a normal distribution with mean 0.1 and standard deviation 0.01. We assumed complete independence between the probability of being vaccination by routine vaccine or by an SIA, as was assumed in the simulation.

We assumed incident case reports by age were available but under-reported at time points 6, 11, 16, and 26 by 95%, 92%, 88%, and 85%, respectively. We assumed case reporting improved over time as the simulated area moves from endemic to elimination settings, similar to country’s realistic improvement is surveillance over time. Age-specific incidence adjusted for under-reporting by assuming 85% underreporting (note this is another source of uncertainty).

The estimated age distribution of immunity (i.e., $\hat{p(a)}$) at time points 6, 11, 16, and 26 was obtained using a simple model where each age cohort’s immunity is estimated based on its experience of immunization (i.e., $\hat{v(a)}$ via routine immunization and SIAs) and its experience of immunity from natural infection (i.e., $\hat{r(a)}$). For example, if 80% of the age cohort was vaccinated and 15% of the age cohort recovered from natural infection, then 83% of the cohort would be estimated to be immune (i.e., $\hat{p\left( a \right)}=1-((1-\hat{r\left( a \right)})(1-\hat{v(a)}$))).

**Supplement S2: Empirical Example**

We evaluated the estimated proportion rubella seropositive from serological data compared to that inferred using demographic and vaccination data for rural Tamil Nadu, India. Rubella serological data was extracted from published work for a study conducted among women ages 1 to 39 years between 1999 and 2000 from rural Vellore, Tamil Nadu, India; the data sample size is 1693 [22]. The published serological data included the total number tested by age and the number tested seropositive by age. The results are displayed as hallow black dots in Supplemental Figure 2.

We additionally inferred the proportion rubella seropositive (i.e., $\hat{p(a)}$) using vaccination data (i.e., routine rubella-containing vaccine coverage) from Rapid Survey on Children 2013-14 [23] to estimate proportion vaccinated by age (i.e., $\hat{v(a)}$) and known rubella epidemiological parameters to estimate proportion recovered from natural infection (i.e., $\hat{r(a)}$).

As of 2000, rubella-containing vaccine (RCV) was only administered in the private-sector. We assumed that any child who received their vaccinations in a private healthcare center received RCV, because the Indian Academy of Pediatrics recommends MMR vaccine for routine use at age 9 months, 15 months, and 4 to 6 years old [24]. The estimated proportion of children who received vaccinations in private healthcare centers for rural Tamil Nadu were extracted from the Rapid Survey on Children [23], and is 13.4%. Private-sector routine RCV began in 1993, when Serum Institute of India first launched India's Measles-Mumps-Rubella (MMR) vaccine. As a result, we assumed private-sector RCV coverage of 13.4% among 12 month olds (with 97% vaccine efficacy) for all age cohorts born between 1993 and 2000. Supplemental Figure 2, bottom right shows the resultant estimated proportion vaccinated by age (i.e., $\hat{v(a)}$) as of 2000.

Given the lack of publically available data on rubella incidence in rural Tamil Nadu, we assumed an age-specific proportion recovered from natural rubella infection (i.e., $\hat{r(a)}$) based on the exponential distribution where 85% of infections occurred by 20 years old (Supplemental Figure 2, bottom right). Discrepancies between the serological profile and inferred profile of seropositivity over age, especially in very young age groups will emerge from uncertainties linked to the two data-streams on vaccinal and natural immunity deployed; thus illustrating the major issue that serological data may be able to address, further detailed in the main text.

**Figure 2:** Estimated age-specific proportion seropositive based on serological data (black dots) and inferred estimate (blue dashed line) for rural Tamil Nadu, 2000. The blue dashed line is inferred from the proportion vaccination ($\hat{v(a)}$) for rural Tamil Nadu and proportion recovered from natural infection ($\hat{r(a)}$), displayed in the bottom right of the figure. We find that the inferred estimates generally over-estimated proportion seropositive among children less than 7 years old, and under-estimated proportion seropositive among individuals ages 10 to 13 years.

**Supplement S3: Serology and spatial patterns of immunity**

Patterns of immunity to measles and rubella may vary spatially [25], and this will interact with patterns of immunity over age. Measles and rubella are at risk of extinction where there are insufficient numbers of susceptible individuals to maintain chains of transmission, and re-introductions are relatively rare, a phenomenon characterized using the concept of a critical community size (CCS) [8, 26-28]. Communities below the CCS are more likely to have experienced sequential years without circulating virus, allowing the accumulation of susceptible individuals through births. These individuals may also have had the opportunity to age in the absence of infection [8], thus modifying patterns of immunity over age. Vaccination increases the community size required to sustain transmission because it reduces the number of susceptible individuals entering the population.

If the history of virus extinction across communities could be known, this might support targeting of control efforts, either spatially (targeting communities known to have experienced susceptible buildup), or across age (targeting individuals known to have lived through years where transmission was absent). However, since the CCS is shaped by susceptible replenishment, vaccination, and re-introductions of the infection via human mobility, it is likely to be both variable across contexts, and hard to characterize using available case notification or vaccination data alone, particularly as under-reporting complicates detection of extinction from case notification data [29].

Despite this complexity, theoretically defined characteristics of the areas at risk of high susceptibility as a result of spatial patterns of extinction could potentially be leveraged in combination with serology to support targeting of control efforts. For example, serological surveys conducted in sites identified as being at risk of high rates of susceptibility (i.e., remote areas or those with small populations potentially prone to local extinction or poor access to vaccinations) could, in theory, be used to identify spatial immunity gaps; however, this type of surveillance has never been conducted and the relative costs and benefits of such efforts are an open area of research.

**Supplement S4: Feasibility and Sustainability of Serological Surveys**

Rigorously conducted, cross-sectional community-based surveys with blood specimen collection require: i) substantial financial resources and time commitment, ii) logistical capacity and skilled personnel to design and conduct a serosurvey that is generalizable to the target population, iii) laboratories and laboratory expertise to perform the serological assays with proper quality control and assurance, and iv) technical expertise in statistical analysis to interpret serological data [30]. For example, biased sampling strategies that are not rigorously planned and executed to ensure representative samples of the target population will result is inaccurate age-specific seroprevalence estimates. Therefore, high levels of community participation and acceptability of blood collection via serological sampling via (e.g. venipuncture or finger prick) are critical and should be ensured through carefully planned community mobilization activities, in light of the limited estimates of refusal rates [31].

Various resources are available to support serosurvey development in the face of these challenges, including international and regional guidelines, such as the World Health Organization (WHO) vaccination coverage survey guidelines [32] (which particularly informs the issue of designing a representative survey), and WHO ‘Guidance on conducting a serosurvey in support of measles and rubella elimination in the WHO European Region’ which speaks to all aspects of serosurveys, although it is targeted towards European countries [33]. However, there remains no international guidance or list of validated laboratory methods for measles and rubella IgG serology; in fact a review of 97 published measles and rubella seroprevalence studies demonstrated that a large range of sample types, assays, equivocal ranges and cut-offs have been employed over a 16-year period of publications [34], which can impact estimation of seroprofiles of immunity [35]. A WHO working group is currently working towards drafting international guidelines on the use of serosurveys in support of measles and rubella elimination.

Despite the potential of serological surveys and some guidance handbooks, no low- or lower-middle income country is currently conducting routine serological surveillance to monitor measles or rubella immunity given the cost as well as logistical and technical challenges. A critical research question is how to make serological surveys sustainable in low-resource settings. To be sustainable, serological surveys will likely need to be nested within existing surveillance systems or regularly conducted household surveys and provide information on multiple infectious diseases (and perhaps non-communicable diseases), including a range of vaccine-preventable and emerging infectious diseases. Before this is feasible, however, field-friendly assays for measles and rubella that provide reliable, accurate and reproducible measures of immunity need to be developed.

***Expanding existing sample collection and surveillance systems to include serology***

Serological surveys may be made more sustainable by integrating them within existing sample collection platforms, with several opportunities to leverage on-going surveillance systems. For example, opportunistic serological surveys can use blood sample remainders from hospitals, prenatal care visits or blood donors, although understanding the potential biases in the external validity these samples is critical. Importantly, existing disease surveillance can be leveraged, adding immunoglobulin G (IgG) antibody testing on serum samples collected as part of the fever-rash surveillance network for measles and rubella which exists in many countries [36]. These sample sources may be augmented by a small random and representative sample of the general population to correct for overrepresentation of specific populations or socio-demographic groups such that the results can be generalized.

Household surveys, such as the Demographic Health Surveys or Expanded Programme on Immunization (EPI) Coverage Surveys, could be extended to include collection of blood or oral fluid samples for serological testing.  Such studies are generally designed to be nationally representative, thus strengthening the ability to extrapolate age-specific seroprevalence estimates to national and subnational levels. However, household surveys are burdensome and adding further activities is likely to be challenging unless relatively simple to incorporate and of high value. An alternative to building on existing surveys or surveillance is to define a network of sentinel surveillance sites. Knowledge of the underlying epidemiology, as well as socio-economic and demographic contexts, could be used to define the optimal locations of sentinel sites.

***Expanding the benefits of serological surveillance: quantitative data and multiplex assays***

Quantitative data, i.e., the antibody concentration or titer in serum, is often used to assess immunity based on a binary test of whether it is above or below a threshold considered protective [37, 38]. However, considerably more insight may be gained from evaluating these data beyond the binary designation of seropositive or seronegative. For instance, understanding the degree to which boosting of immunity through exposure to wild-type rubella virus plays a role in maintaining rubella immunity is an increasingly important question. Seasonal cycles of boosting of rubella immunity suggest that this may be important in maintaining population immunity [39].

Beyond rubella and measles, the sustainability of serological surveys may be strengthened by including other vaccine-preventable and emerging infectious diseases [40]. Multiplexed assays that permit simultaneous detection of multiple antigens could be performed to broaden the scope of serosurveys. Such assays can measure antibodies to vaccine-preventable diseases (e.g. measles, mumps, rubella, polio and varicella viruses as well as tetanus, diphtheria and pertussis antigens), neglected tropical diseases (e.g. ascaris, schistosomiasis, and trachoma), vector-borne diseases (e.g. malaria and dengue viruses), and waterborne pathogens (e.g. giardia, enterotoxigenic *Escherichia coli*, and cholera). These assays can be leveraged in countries that administer combined vaccines, i.e., measles-rubella (MR), measles-mumps-rubella (MMR), or measles-mumps-rubella-varicella (MMR) vaccines to estimate vaccine coverage [41-43]. Several efforts are underway to optimize multiplex assays to measure IgG antibodies to measles and rubella viruses as well as other vaccine-preventable and emerging infectious diseases. There is ongoing effort to transfer this technology to several laboratories around the world to perform multiplexed serological assays [44, 45]. Although currently expensive, testing biomarkers beyond vaccine-preventable diseases expands the relevance of serological surveillance and may make it a more sustainable tool for countries to deploy [40].

***Standardizing specimen collection, testing and interpretation of serological results***

Expanding existing surveillance activities to include serology must be compatible with available resources and laboratory capacity. In resource poor settings, a single laboratory with limited personnel may be a major barrier to conducting large-scale or frequent serological surveys. Improvements in commercially available assays, development of standard laboratory protocols, and establishment of reporting requirements would lessen the burden on individual laboratories and facilitate comparisons of results across settings and time. In-country laboratories usually must use commercially available enzyme immunoassays, which are often less sensitive than the gold standard assays, such as plaque reduction neutralization tests. Standard cut-offs to define seroprotection or seropositivity (depending on the objectives) need to be defined and ideally verified during testing using a reference standard [46, 47]. Establishment of standard laboratory testing requirements would be a significant step forward. Options include establishing a formal external quality assessment (EQA) system as conducted in UK and Europe [48, 49] or establishing quality assurance requirements such as validation testing a subsample with the gold standard assay, running an international reference standard or a standard serum panel on a subset of samples, and establishing expectations for duplicate testing or the proportion of samples that should be randomly retested. Consensus on minimum reporting requirements are also needed and should include reporting of relevant quality control indicators (coefficient of variation, interclass correlation coefficient, results for international reference standards or standard serum panels), treatment of equivocal results, and cutoffs used in the analysis. Lastly, the tradeoffs for using less invasive sampling (e.g., oral fluid [50], finger-prick, or potentially fecal samples [51] pending further research on measles and rubella mucosal immunity) or point-of-care tests should be further evaluated as the costs of lower sensitivity and specificity may be offset by the benefits in acceptability, speed and ease of deployment.

**Works Cited**

1. Klepac P, Caswell H. The stage-structured epidemic: linking disease and demography with a multi-state matrix approach model. Theor Ecol-Neth **2011**; 4:301-19.

2. Klepac P, Pomeroy LW, Bjornstad ON, Kuiken T, Osterhaus ADME, Rijks JM. Stage-structured transmission of phocine distemper virus in the Dutch 2002 outbreak. P Roy Soc B-Biol Sci **2009**; 276:2469-76.

3. Metcalf CJE, Lessler J, Klepac P, Morice A, Grenfell BT, Bjornstad ON. Structured models of infectious disease: Inference with discrete data. Theor Popul Biol **2012**; 82:275-82.

4. Metcalf CJE, Lessler J, Klepac P, Cutts F, Grenfell BT. Impact of birth rate, seasonality and transmission rate on minimum levels of coverage needed for rubella vaccination. Epidemiol Infect **2012**; 140:2290-301.

5. Bjornstad ON, Finkenstadt BF, Grenfell BT. Dynamics of measles epidemics: Estimating scaling of transmission rates using a time series SIR model. Ecol Monogr **2002**; 72:169-84.

6. Finkenstadt BF, Grenfell BT. Time series modelling of childhood diseases: a dynamical systems approach. J Roy Stat Soc C-App **2000**; 49:187-205.

7. Glass K, Xia Y, Grenfell BT. Interpreting time-series analyses for continuous-time biological models-measles as a case study. J Theor Biol **2003**; 223:19-25.

8. Metcalf CJE, Munayco CV, Chowell G, Grenfell BT, Bjornstad ON. Rubella metapopulation dynamics and importance of spatial coupling to the risk of congenital rubella syndrome in Peru. J R Soc Interface **2011**; 8:369-76.

9. Metcalf CJE, Bjornstad ON, Ferrari MJ, et al. The epidemiology of rubella in Mexico: seasonality, stochasticity and regional variation. Epidemiol Infect **2011**; 139:1029-38.

10. Keeling MJ, Rohani P, Grenfell BT. Seasonally forced disease dynamics explored as switching between attractors. Physica D **2001**; 148:317-35.

11. Mossong J, Hens N, Jit M, et al. Social contacts and mixing patterns relevant to the spread of infectious diseases. Plos Med **2008**; 5:381-91.

12. Horby P, Pham QT, Hens N, et al. Social contact patterns in Vietnam and implications for the control of infectious diseases. Plos One **2011**; 6:e16965.

13. DeStefano F, Haber M, Currivan D, et al. Factors associated with social contacts in four communities during the 2007-2008 influenza season. Epidemiol Infect **2011**; 139:1181-90.

14. Edmunds WJ, Gay NJ, Kretzschmar M, Pebody RG, Wachmann H, Project E. The pre-vaccination epidemiology of measles, mumps and rubella in Europe: implications for modelling studies. Epidemiol Infect **2000**; 125:635-50.

15. Cutts FT, Abebe A, Messele T, et al. Sero-epidemiology of rubella in the urban population of Addis Ababa, Ethiopia. Epidemiol Infect **2000**; 124:467-79.

16. Lessler J, Metcalf CJE. Balancing evidence and uncertainty when considering rubella vaccine introduction. Plos One **2013**; 8:e67639.

17. Waaijenborg S, Hahné SJM, Mollema L, et al. Waning of maternal antibodies against measles, mumps, rubella, and varicella in communities with contrasting vaccination coverage. The Journal of infectious diseases **2013**; 208:10-6.

18. Nicoara C, Zach K, Trachsel D, Germann D, Matter L. Decay of passively acquired maternal antibodies against measles, mumps, and rubella viruses. Clin Diagn Lab Immun **1999**; 6:868-71.

19. Lessler J, Metcalf CJE, Grais RF, Luquero FJ, Cummings DAT, Grenfell BT. Measuring the performance of vaccination programs using cross-sectional surveys: A likelihood framework and retrospective analysis. Plos Med **2011**; 8.

20. Cutts FT, Izurieta HS, Rhoda DA. Measuring coverage in MNCH: Design, implementation, and interpretation challenges associated with tracking vaccination coverage using household surveys. Plos Med **2013**; 10.

21. Boulianne N, De Serres G, Ratnam S, Ward BJ, Joly JR, Duval B. Measles, mumps, and rubella antibodies in children 5-6 years after immunization: effect of vaccine type and age at vaccination. Vaccine **1995**; 13:1611-6.

22. Vynnycky E, Adams EJ, Cutts FT, et al. Using seroprevalence and immunisation coverage data to estimate the global burden of congenital rubella syndrome, 1996-2010: A systematic review. Plos One **2016**; 11:e0149160.

23. Ministry of Women and Child Development - Government of India. Rapid Survey on Children (2013-14): India and State Fact Sheets. Available at: <http://wcd.nic.in/acts/rapid-survey-children-rsoc-2013-14>. Accessed January 4 2017.

24. Indian Academy of Pediatrics. IAP Recommendations and Guidelines. Available at: [http://www.iapindia.org/page.php?id=129#](http://www.iapindia.org/page.php?id=129). Accessed January 13 2017.

25. Hens N, Abrams S, Santermans E, et al. Assessing the risk of measles resurgence in a highly vaccinated population: Belgium anno 2013. Eurosurveillance **2015**; 20.

26. Bartlett MS. The Critical Community Size for Measles in the United-States. J R Stat Soc Ser a-G **1960**; 123:37-44.

27. Bartlett MS. Measles Periodicity and Community Size. J R Stat Soc Ser a-G **1957**; 120:48-70.

28. Metcalf CJE, Cohen C, Lessler J, et al. Implications of spatially heterogeneous vaccination coverage for the risk of congenital rubella syndrome in South Africa. J R Soc Interface **2013**; 10.

29. Gunning CE, Wearing HJ. Probabilistic measures of persistence and extinction in measles (meta)populations. Ecol Lett **2013**; 16:985-94.

30. Cutts FT, Hanson M. Seroepidemiology: an underused tool for designing and monitoring vaccination programmes in low- and middle-income countries. Trop Med Int Health **2016**.

31. McNaghten AD, Herold JM, Dube HM, St Louis ME. Response rates for providing a blood specimen for HIV testing in a population-based survey of young adults in Zimbabwe. Bmc Public Health **2007**; 7.

32. World Health Organization. Monitoring and Assessing Immunization Systems. Available at: <http://www.who.int/immunization/monitoring_surveillance/routine/coverage/en/index2.html>. Accessed Feburary 5 2018.

33. WHO Regional Office for Europe. Guidance on conducting serosurveys in support of measles and rubella elimination in the WHO European Region. **2013**.

34. Dimech W, Mulders MN. A review of testing used in seroprevalence studies on measles and rubella. Vaccine **2016**; 34:4119-22.

35. Hull SC, Caplan AL. The case for vaccinating boys against human papillomavirus. Public Health Genomics **2009**; 12:362-7.

36. Mulders MN, Rota PA, Icenogle JP, et al. Global measles and rubella laboratory network support for elimination goals, 2010-2015. Morbidity and Mortality Weekly Report **2016**; 65:438-42.

37. Moss WJ, Scott S. The immunological basis for immunization series : module 7: measles - Update 2009. In: Immunization Vaccines and Biologicals, World Health Organization, eds, **2009**.

38. Best JM, Reef S. Immunological basis for immunization: module 11: rubella. In: Immunization Vaccines and Biologicals, World Health Organization, eds, **2008**.

39. Rosenblatt LS, Shifrine M, Hetherington NW, Paglierioni T, Mackenzie MR. A Circannual rhythm in rubella antibody-titers. J Interdiscipl Cycle **1982**; 13:81-8.

40. Metcalf CJE, Farrar J, Cutts FT, et al. Use of serological surveys to generate key insights into the changing global landscape of infectious disease. The Lancet **2016**; 388:728-30.

41. Goeyvaerts N, Hens N, Theeten H, Aerts M, Van Damme P, Beutels P. Estimating vaccination coverage for the trivalent measles-mumps-rubella vaccine from trivariate serological data. Stat Med **2012**; 31:1432-49.

42. Wood JG, Goeyvaerts N, MacIntyre CR, Menzies RI, McIntyre PB, Hens N. Estimating vaccine coverage from serial trivariate serologic data in the presence of waning immunity. Epidemiology **2015**; 26:381-9.

43. Altmann D, Altmann K. Estimating vaccine coverage by using computer algebra. Ima J Math Appl Med **2000**; 17:137-46.

44. Lammie PJ, Moss DM, Goodhew EB, et al. Development of a new platform for neglected tropical disease surveillance. Int J Parasitol **2012**; 42:797-800.

45. Smits GP, van Gageldonk PG, Schouls LM, van der Klis FRM, Berbers GAM. Development of a bead-based multiplex immunoassay for simultaneous quantitative detection of IgG serum antibodies against measles, mumps, rubella, and varicella-zoster virus. Clin Vaccine Immunol **2012**; 19:396-400.

46. Diaz-Ortega JL, Forsey T, Clements CJ, Milstien J. The Relationship between dose and response of standard measles-vaccines. Biologicals **1994**; 22:35-44.

47. Anderson RM, May RM. Infectious Diseases of Humans: Dynamics and Control. Oxford University Press, **1991**.

48. Kafatos G, Andrews N, Mcconway KJ, et al. Estimating seroprevalence of vaccine-preventable infections: is it worth standardizing the serological outcomes to adjust for different assays and laboratories? Epidemiol Infect **2015**; 143:2269-78.

49. European Centre for Disease Prevention and Control. External quality assurance scheme for diphtheria diagnostics 2012. Stockholm, **2013**.

50. Nigatu W, Samuel D, Cohen B, et al. Evaluation of a measles vaccine campaign in Ethiopia using oral-fluid antibody surveys. Vaccine **2008**; 26:4769-74.

51. Vetvik H, Grewal HMS, Haugen IL, Ahren C, Haneberg B. Mucosal antibodies can be measured in air-dried samples of saliva and feces. J Immunol Methods **1998**; 215:163-72.
